# Novel Bat‐Monitoring Dataset Reveals Targeted Foraging With Agricultural and Pest Control Implications

**DOI:** 10.1002/ece3.70819

**Published:** 2025-01-15

**Authors:** Brian Lee, Samantha Sambado, D. Nākoa Farrant, Anna Boser, Kacie Ring, David Hyon, Ashley E. Larsen, Andrew J. MacDonald

**Affiliations:** ^1^ Bren School of Environmental Science & Management University of California Santa Barbara California USA; ^2^ Department of Ecology, Evolution, and Marine Biology University of California Santa Barbara California USA

**Keywords:** agriculture, agroecology, bat ecology, California's Central Valley, ecosystem services, mosquitos

## Abstract

Quantifying ecosystem services provided by mobile species like insectivorous bats remains a challenge, particularly in understanding where and how these services vary over space and time. Bats are known to offer valuable ecosystem services, such as mitigating insect pest damage to crops, reducing pesticide use, and reducing nuisance pest populations. However, determining where bats forage is difficult to monitor. In this study, we use a weather‐radar‐based bat‐monitoring algorithm to estimate bat foraging distributions during the peak season of 2019 in California's Northern Central Valley. This region is characterized by valuable agricultural crops and significant populations of both crop and nuisance pests, including midges, moths, mosquitos, and flies. Our results show that bat activity is high but unevenly distributed, with rice fields experiencing significantly elevated activity compared to other land cover types. Specifically, bat activity over rice fields is 1.5 times higher than over any other land cover class and nearly double that of any other agricultural land cover. While irrigated rice fields may provide abundant prey, wetland and water areas showed less than half the bat activity per hectare compared to rice fields. Controlling for land cover type, we found bat activity significantly associated with higher flying insect abundance, indicating that bats forage in areas where crop and nuisance pests are likely to be found. This study demonstrates the effectiveness of radar‐based bat monitoring in identifying where and when bats provide ecosystem services.

## Graphical abstract

This study uses a weather‐radar‐based monitoring algorithm to track bat foraging in California's Northern Central Valley, revealing high bat activity concentrated around rice fields. Bat presence was significantly linked to areas of high mosquito abundance and West Nile Virus transmission risk, highlighting the potential of bats to provide ecosystem services in agriculture and public health.

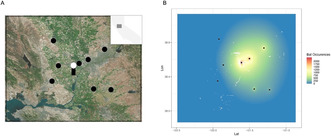

## 1 Introduction

Biodiversity loss, urbanization, and climate change are occurring globally, with consequences for ecosystem services at varying spatial scales (Elmhagen, Eriksson, and Lindborg 2015; Mooney et al. 2009; Prangel et al. 2023). Yet, the quantification of services tied to the activity of specific mobile taxa like pollinators or pest control agents remain elusive due to the difficulty of tracking these dynamic taxa in space and time (Charbonnier et al. 2021; Hanley et al. 2015; Kunz et al. 2011).

Bats are vital to ecosystems by offering essential services such as pollination, fertilization through their guano, and natural pest control. Their behavior and diet help reduce populations of both crop and nuisance pests, thereby decreasing the reliance on pesticides (Betke et al. 2008; Boyles et al. 2011; Federico et al. 2008; Kasso and Balakrishnan 2013; Kunz et al. 2011; Reiskind and Wund 2009). Prior studies have estimated the pest control value of insectivorous bats, but these estimates rely on exclusion experiments, acoustic monitoring, or limited diet studies of small bat populations, which are then extrapolated to larger areas (Boyles et al. 2011; Kasso and Balakrishnan 2013; Kunz et al. 2011). Such snapshots, while valuable for understanding dietary niches, fall short in capturing the full spectrum of bat foraging behavior across large landscapes and entire foraging seasons.

California's Northern Central Valley is an important landscape to understand where bats are foraging due to the potential impacts bats could have for both the agriculture and pest control. Compared to the rest of California, the Northern Central Valley has extensive irrigated agriculture that has created habitat with an abundance of insects and water sources allowing bats, particularly the Mexican free‐tailed bats ( *Tadarida brasiliensis* ), to maintain robust populations (Smith et al. 2021). With an estimated population of 250,000–500,000 spread across multiple large roosts, Mexican free‐tailed bats in the Sacramento Valley primarily consume moths, water boatman, beetles, flies, midges, and plant bugs. Diet analysis indicate temporal patterns in their consumption with flies, midges, and mosquitos accounting for 85% of the total volume of their diet in April and May (Long et al. 1998). These bat populations could perform critical ecosystems services related to pest control and economic outcomes in California's croplands. For example, the Central Valley hosts large populations of nuisance pests due to a combination of favorable landscapes, such as standing water that supports insect breeding, and the overlapping activity of pests like midges, flies, and mosquitos around agricultural workers (Barber, Schleier, and Peterson 2010; Reisen et al. 2009) The pest prevalence poses significant challenges for agriculture, which is threatened by the direct loss of crops through arthropod pests (Oerke 2006), motivating the use of millions of tons of insecticides each year (CA PUR, 2020). Although bats are assumed to be positively involved with these systems, there has not been a rigorous effort to map where bats preferentially forage and if that foraging activity overlaps with high‐value crops or an abundance of infected mosquitos.

While there is a population of ecosystem providers and a clear demand for services like controlling agricultural pest populations, the effectiveness of bats in providing these services largely depends on their foraging location. If bats forage primarily in the pockets of natural riparian woodlands, they may still provide a valuable ecosystem function, but the benefits for crops and nuisance pest suppression may be limited. Alternatively, if bats forage in valuable crop areas that are particularly conducive to crop and nuisance pests, their economic benefits to human communities could be substantial. Yet, as with many ecosystem services tied to highly mobile taxa, capturing the spatiotemporal patterns of foraging is extremely difficult. Previous sampling of bat populations required intensive field studies (Kurta and Whitaker 1998; Tuneu‐Corral et al. 2020; Whitaker and Rissler 1992), which often resulted in only population numbers at a given location, rather than a distribution of bat activity beyond roosting locations. Estimating population numbers and mapping where bats forage—both metrics important for quantifying ecosystem services—were limited by previous approaches (Horn, Arnett, and Kunz 2008; Tuneu‐Corral et al. 2020).

Here, we leverage a new bat‐monitoring algorithm to understand the distribution of bat activity across a heterogeneous landscape. The algorithm, named Bat‐Aggregated Time Series (BATS), is a novel data workflow that relies on an artificial neural network to identify Mexican free‐tailed bat presence in Doppler weather radar (Lee et al. 2024). National Oceanic and Atmospheric Administration's (NOAA) Next Generation Weather Radar (NEXRAD) system is increasingly used to track migratory birds and other flying species over long distances (Ansari et al. 2018; Chilson et al. 2012; Chowdhury et al. 2016) and can also be used to track the presence of bats over time across a large study area (Gauthreaux and Diehl 2020; Tielens et al. 2021). We focus on California's Northern Central Valley, a region of particularly high economic agricultural value with a persistent population of crop and nuisance pests. Within this region, we map where bat activity is occurring to assess the potential ecosystem services bats may provide by bringing in climate, land cover, and insect data—using mosquitos as a proxy for other flying insect populations—to address the following questions:

1. How does bat activity vary across crop types and land covers?
2. Does bat activity overlap with areas that have a higher abundance of nuisance pests?

## 2 Materials and Methods

### 2.1 Study Area

The Sacramento‐San Joaquin Delta in California's Northern Central Valley is an important agricultural area of the United States. It is a highly heterogeneous landscape, featuring a fertile agricultural valley surrounded by foothills and mountains covered in natural vegetation. More than 250 different crop types are grown throughout the valley, producing 13% of the nation's food with an estimated economic output of $51.1 billion per year (CDFA, 2020).

**FIGURE 1:**
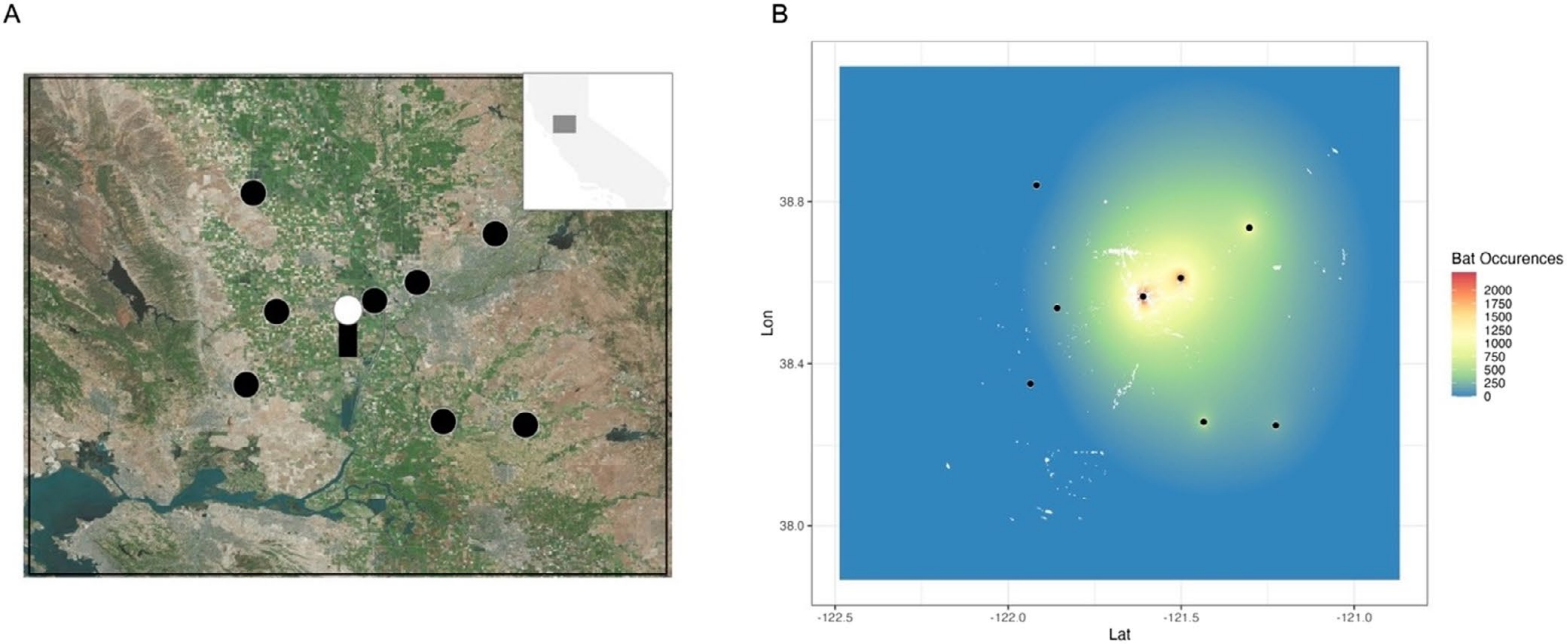
Aggregated bat presence layers using the Bat‐Aggregated Time Series (BATS) algorithm. (a) The study is in California's Northern Central Valley, with known bat roosts represented in large black circles. In the center of our study area is the NEXRAD Doppler Radar Tower KDAX. Image *Source:* Bing Virtual Earth. (b)The final corrected bats data aggregated across the season, which is referred to as bat activity throughout the text.

For this paper, we focus on a 140 × 120 km study area surrounding the National Weather Service NEXRAD Tower KDAX, located 7 km southeast of Davis, California (Figure 1a). We chose an area with eight, large known bat colonies, including the Yolo and Cosumnes bat colonies. This region is characterized by its diverse agricultural landscape, which includes a variety of crops that can benefit from the pest control services provided by bats. In addition to agricultural pests, nuisance pests such as flies, midges, and mosquitos are concentrated in this region, highlighting the value of knowing where bats are foraging, and if their activity overlaps areas with arthropods that impact agriculture. To capture the full potential effects of bats, we downloaded and analyzed data focusing on the bat foraging season of May to October of 2019 when bats are most active, and their impact on both agriculture and mosquito populations are potentially most significant (Long et al. 1998).

### 2.2 Bat Presence

We mapped bat presence to assess the distribution of bat foraging activity. Here, “bat presence” refers to the detection of a group of bats within radar data, defined not by a precise count of individuals but rather by a relative measure of density sufficient to register in the radar signal. This density‐based presence was quantified per pixel; for instance, if a large enough group of bats foraged over a field and was detected by radar five times in one evening, that pixel would receive a value of five. Although we lack exact on‐the‐ground numbers, this radar‐based approach allowed us to map relative foraging intensity and capture a detailed spatial distribution of bat activity across the study area. Bat presence data are derived from NEXRAD Doppler weather data accessed through NOAA's Open Data program hosted on Amazon Web Services (Ansari et al. 2018; NOAA National Weather Service 1991). The nearest NEXRAD radar tower to our study region, Tower KDAX, collects radar data every 4–10 min over our entire targeted area. We downloaded data for 152 evenings between the bat foraging months of May through October, a total of 19,169 radar scenes, with an average of 126 scenes processed per evening, between the hours of 6:00 PM PST to 6:00 AM PST. These data are processed within the BATS data workflow, which downloads, pre‐processes, and classifies pixels as binary bat presence‐absence using a feed‐forward neural network described in Lee et al. (2024). Data pre‐processing includes mapping the radar data to a Cartesian Grid with a resampled resolution of 70 × 70 m pixels (Helmus and Collis 2016). Details of the additional post processing of the data can be found in Appendix A SI.1. For this study, we created a single aggregated layer highlighting bat activity from the 2019 foraging season across our study area. To facilitate a comprehensive analysis and comparison, we converted the bat occurrence data from a per‐pixel basis to a standardized unit of bat occurrence per hectare per night, considering the entire 152‐night foraging season (Figure 1b). This conversion allows for a more uniform and detailed understanding of bat activity patterns over the study period.

To account for the natural aggregation of bats at roosts and the consequent higher observed presence in the vicinity—largely due to bats departing and returning rather than foraging—we applied an exponential correction akin to an inverse distance weighting that involves a decay function with distance. Details of the exponential correction can be found in Appendix A SI.1.

### 2.3 Land Cover and Crop Type Analysis

To assess relative bat activity over different land cover and crop types, we used two datasets to produce a comprehensive map of natural and agricultural land cover in the study region. For natural land cover types, we used the 30 m vegetation raster “FVEG” generated for the Fire and Resource Assessment Program based on the best available land cover data from 1990 to 2014 (CALFIRE‐FRAP, 2015). To map cropland types, we leveraged Land IQ's statewide shapefile that distinguishes individual fields and 50 crop types with 97% accuracy for the 2019 water year (Land IQ, 2019).

**FIGURE 2:**
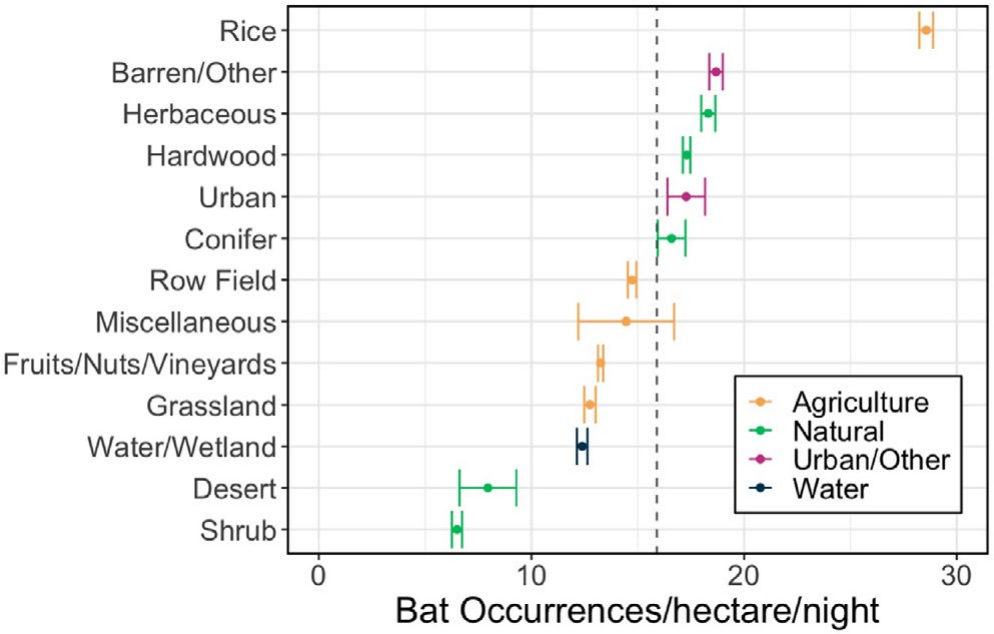
The area‐weighted average bat occurrences per hectare per night within each land cover type. Error bars reflect the 95% confidence interval. Bat occurrences were highest over rice fields and more than 1.5 times higher than any other land cover type. Most natural and urban land cover types had above‐average bat densities, but rice was the only agricultural land cover with an above‐average bat density.

The combined land cover layer consisted of 56 land cover categories derived from the two original datasets which were regrouped into 13 land cover classes (i.e., barren/other, conifer, desert, fruits/nuts/vineyards, grassland, hardwood, herbaceous, rice, row/field crops, shrub, water/wetland, urban, and miscellaneous) and four general land cover types (i.e., agricultural, natural, urban/other, or water) (Figure 2). The “miscellaneous” land cover type is primarily areas that “FVEG” identified as agriculture in 2015 but were not labeled as cropland in the more accurate cropland data from 2019. The miscellaneous land cover includes field margins as well as areas that were potentially fallowed or used for other agricultural purposes. The final land cover types distinguish agricultural, natural, and urban/other land uses with further subcategories associated with vegetation height for functional purposes related to bat activity. Further details regarding land cover and crop type classification can be found in Appendix A S.2.

We extracted the total bat occurrences over each parcel in the study area during the foraging season, using parcel boundaries defined by LandIQ, which delineates fields based on land use validated using high resolution remote sensing imagery (NAIP). The extraction was performed on the bat presence raster that was exponentially corrected for the distance of a pixel from a roost (Figure 1b). Bat occurrences per pixel were calculated within each parcel. Parcels were grouped by land cover class and the area‐weighted average bat occurrences per hectare in each land cover class was calculated (Appendix A S.2). The area‐weighted average bat occurrences per hectare across the entire study region was similarly calculated.

### 2.4 Mosquito Abundance as a Proxy for Other Aerial Pests

In the Sacramento‐San Joaquin Delta region, vector control agencies systematically survey mosquito populations, and this field data from the Mosquito and Vector Control Association of California (VectorSurv, 2023), has been used to validate modeled mosquito abundance through thermal performance curves (MacDonald et al. 2024). Although mosquitos represent only a small portion of bat diets, other flying taxa—such as flies, midges, and moths—constitute a significant part of their diet and follow similar thermal performance curves. Therefore, we use high resolution mosquito surveillance data as a proxy for the abundance of other important flying taxa in bat diets. To model the relationship between bat activity and insect prey densities—using mosquito abundance as a proxy—we first needed to produce a spatially explicit estimate of mosquito abundance across our landscape of interest (Appendix A S.3). Here we use the approach of Boser et al. (2021) to model mosquito abundance in the peak summer season (April–September) from high resolution (70 m) remote sensing of temperature applied to the temperature‐dependent, trait‐based models of *Culex tarsalis* mosquitos by Shocket et al. (2020) and Mordecai et al. (2013). This approach has been independently validated against field vector surveillance data in the same region (MacDonald et al. 2024), showing a strong positive relationship between modeled abundance and surveillance‐based mosquito abundance in the field.

We then pair our estimated mosquito abundance with bat activity, aggregating each to 1 km resolution pixels, to obtain average mosquito abundance and average bat activity in each pixel. We aggregated mosquito abundance in 1 km pixels due to the mobility of mosquitos, which can disperse up to a kilometer or more in a single day (Hamer et al. 2014; Reisen and Lothrop 1995), to estimate average abundance across that range. We then ran a generalized additive model (GAM) predicting bat activity with mosquito abundance—as a proxy for flying insect prey densities—(i.e., do bats forage more where insect prey densities is predicted to be high?) with cubic spline smoothing terms to allow for nonlinearity in the relationship. We additionally control for land use type and other environmental factors that may influence bat foraging activity, including average nighttime light emissions and area of irrigated agriculture (Azam et al. 2016; Kilpatrick, LaDeau, and Marra 2007; Kovach and Kilpatrick 2018). Finally, to account for spatial autocorrelation, we include Gaussian Processing smoothing spline terms for latitude and longitude of each pixel.

## 3 Results

Within our study period, we observed high variability in bat activity across the 16,800 km^2^ area (Figure 1). During the entirety of the 2019 foraging season, bats were detected in 95% of this area. The highest activity recorded equates to approximately 99.77 occurrences per hectare per night (7431 occurrences in a 0.49 ha pixel over 152 nights). This particular area was active in 38% of the 19,169 radar scenes, indicating a hotspot of bat activity.

### 3.1 Bats Activity Was Unevenly Dispersed Across Land Cover Types

Bat activity exhibited distinct patterns across different land cover types (Figure 2), with an average of 15.89 occurrences per hectare per night across all land cover types when normalized across the 152 nights in our study period. The highest average detections occur over rice fields, with an average of 28.56 occurrences per hectare per night, which was 1.5 times higher than the barren/other land cover class that had the next highest bat activity per hectare. Other land cover classes with bat activity higher than the average bat activity in the entire study region included barren landscapes, herbaceous terrains, coniferous forests, hardwood forests, and urban areas. In contrast, natural water bodies and wetlands showed lower levels of bat activity, averaging 12.39 occurrence per hectare per night. Desert and shrub areas, which encompass < 1% and 6% of the total study area, respectively, recorded lowest bat activity with averages of 7.95 and 6.50 occurrences per hectare per night, respectively. Additional information regarding bat activity over other land types can be found in Appendix A SI.2.

### 3.2 Bat Activity Is Higher Where Nuisance Pest Density Is Elevated

Bat activity increases significantly with mosquito abundance, or pest densities, as suggested by the GAM model. As modeled mosquito abundance increases, we predict a significant positive relationship between bat activity (Table 1 and Appendix SI.3).

**TABLE 1:** GAM models predicting bat activity with estimated mosquito abundance—proxy for nuisance pest densities—aggregated at 1 km while controlling for land cover type, nighttime lights, and area of irrigated water.

	Bat activity		
Predictors	Estimates	CI	*p*
(Intercept)	1445.52	1401.01–1490.03	< 0.001
Nighttime lights	68.15	59.89–76.40	< 0.001
Area irrigated	10.25	−4.59–25.08	0.176
LULC: Barren	67.98	7.32–128.65	0.028
LULC: Conifer	−250.89	−341.25–−160.52	< 0.001
LULC: Hardwood	−43.56	−94.39–7.28	0.093
LULC: Herbaceous	12.44	−36.17–61.04	0.616
LULC: Shrub	−92.95	−151.69–−34.22	0.002
LULC: Urban	−109.31	−159.79–−58.83	< 0.001
LULC: Water/Wetland	110.03	56.15–163.91	< 0.001
LULC: Fruits/Nuts/Vineyards	−185.57	−234.79–−136.35	< 0.001
LULC: Row Crops	−91.78	−140.78–−42.78	< 0.001
LULC: Grassland	−176.93	−230.96–−122.89	< 0.001
LULC: Rice	114.54	58.62–170.47	< 0.001
Smooth term: mosquito abundance			< 0.001
Smooth term: Lat./Long.			< 0.001
Observations	26,220		
*R* ^2^	0.716		

## 4 Discussion

Understanding the distribution of ecosystem service providers is crucial for conserving the valuable services they provide to humans and ecological communities. Leveraging new, high resolution bat‐monitoring technology, we quantify the spatial extent of bat activity and demonstrate that potential bat‐provisioned ecosystem services are greater than one might expect if assuming uniform foraging activity. We provide three main contributions: (1) we show bats are highly active over rice fields, but surprisingly less so over wetlands and open water, (2) beyond rice, bats are not otherwise foraging at above‐average rates in other agricultural land covers and are more likely to be active over natural land, (3) bat activity is positively associated with higher aerial pest densities—modeled as mosquito abundance.

Investigating the association between bats and agricultural pests has been a topic of interest for decades due to its potential economic relevance (Boyles et al. 2011; Cleveland et al. 2006; Kunz et al. 2011; Rodríguez‐San Pedro et al. 2020). Numerous scholars have highlighted the value of bats as predators of agricultural pests (Kahnonitch, Lubin, and Korine 2018; Louis et al. 2022; Monck‐Whipp et al. 2018; Olimpi and Philpott 2018; Polyakov, Weller, and Tietje 2019; Puig‐Montserrat et al. 2015; Russo, Bosso, and Ancillotto 2018), yet quantifying where and to what extent bats preferentially fly and forage over agriculture has remained a challenge. Using a recently developed neural network algorithm for monitoring bat populations (Lee et al. 2024), we find that rice fields, which are often flood irrigated, are particularly high bat activity areas (Figure 2). Surprisingly, we find activity over water and wetlands to be roughly half of the foraging activity over rice fields. Rice fields in our study were generally large patches of homogenous land covers compared to small fragments of water and wetlands. Thus, there are two possible explanations for our results—first, the community of prey may differ between wet natural habitat and flooded rice fields (Kasso and Balakrishnan 2013; Monck‐Whipp et al. 2018; Park 2015). Second, there could be landscape effects of contiguous rice fields that enable larger or more stable insect populations (Gonsalves et al. 2013; Louis et al. 2022; Norris 2004; Puig‐Montserrat et al. 2015). Bats' preference for rice fields during the summer months suggests a few ecological factors may be attracting bats. Rice fields in California's Northern Central Valley are typically flooded from spring through mid‐summer, which may create an ideal foraging environment (Linquist et al. 2015). The standing water can support a diverse insect community by providing breeding sites for aquatic and semi‐aquatic insects, which bats can exploit (Pathak 1968). Furthermore, the standing water may act as a water source for bats (Adams and Hayes 2008; Mas et al. 2021; Park 2015; Smith et al. 2021). Rice pests, such as armyworm ( *Spodoptera frugiperda* ) and rice water weevil (*Lissorphoptrus oryzophilus*), are active during our study period and potentially serve as a rich food source for foraging bats (UCANR Pest Management). The life cycles of these pests coincide with the rice growing season during the summer months, leading to a higher prey density in these areas (UC Davis Army Worm Monitoring). The combination of these factors likely contributes to the observed high activity levels of bats over rice fields.

Beyond rice, bats in our study region did not have strong affinities to other agricultural land covers and were more active over natural land types (Figure 2). Intensive agriculture has been attributed as a direct and indirect threat to bat populations. Agriculture can directly affect bats through frequent applications of pesticides and other agrochemicals that could be toxic to bats, which could reduce bat activity over crops (Bayat et al. 2014; Kahnonitch, Lubin, and Korine 2018). Converting natural vegetation to agriculture could also reduce suitable roosting habitat for bats (Park 2015). Indirectly, the use of pesticide reduces prey abundances, which could deter the aggregation of bats over a particular agricultural parcel (Olimpi and Philpott 2018). The lack of pesticide applications in natural land covers such as conifers, herbaceous and hardwood types could create suitable habitat for prey, which could explain the above‐average bat activity over these areas as we observed here. This finding aligns with prior results from Olimpi and Philpott 2018 that found total bat activity to be five times greater in natural than agricultural (organic and conventional) land. Natural land cover such as conifers, herbaceous and hardwood types may also offer shelter from wind and predators, but bat behavioral preferences (i.e., foraging, resting, transiting) within these natural lands could be explored further with targeted on‐the‐ground studies involving acoustic or netting surveys. Whether bat activity behavior is driven by resource or shelter needs, bats are unevenly distributed throughout the landscape and their activity patterns may be better assessed through evaluation of local land management practices.

We additionally find evidence that bats disproportionately forage in areas where pest densities are regionally high and where pest control is most critically needed. The Central Valley hosts large populations of flying pests due to a combination of favorable landscapes, such as standing water that supports insect breeding, plentiful food sources in the form of widespread agricultural activity, and the opportunity for nuisance pests to target agricultural workers (Baldwin et al. 2013). Standing water from irrigation or natural bodies of water may attract bat populations in search of concentrated prey sources (Kunz et al. 2011; Park 2015). In our analysis of the relationship between bat activity and mosquito abundance—as a proxy for aerial pests—we found a significant positive association between bat activity and mosquito abundance (Table 1). However, mosquitos are not a primary food source for bats, and their broader diet includes moths and other agricultural pests. For example, Mexican free‐tailed bats have been shown to shift their diet seasonally, consuming mosquitoes, midges, and flies early in the season, but targeting pests such as fall armyworm and other moths later as these species become more abundant (Long et al. 1989). This seasonal dietary shift underscores the broader ecological role bats play in pest control, particularly when key agricultural pests emerge in large numbers (UC Davis Army Worm Monitoring). While bats may consume mosquitoes opportunistically, their foraging patterns are likely tied to the availability of high‐energy prey such as moths, which represent a greater return on energy investment. Extending this work to analyze bat activity in relation to other pest populations across the summer season would provide a more comprehensive understanding of their role in agricultural pest control. Efforts to quantify changes in bat activity and pest densities at broader scales could help refine estimates of bats' contributions to pest control in agricultural landscapes.

While our study has made significant strides in utilizing a novel method to detect bat activity across a large landscape and throughout the bat foraging season, it underscores several challenges that may be explored. A key challenge lies in the machine learning model's capacity to distinguish bat signals amidst urban clutter and various noise disturbances present in the data. The intricate nature of diverse landscapes, characterized by tall structures and uneven terrain, poses a substantial obstacle in precisely monitoring the movements of bats. For this study, major surface elements like sizeable buildings and terrain facing the radar, which often led to incorrect positive classifications, were eliminated through the application of a masking layer. Additionally, we cannot conclusively determine whether radar‐detected bat activity corresponds to foraging or transiting behavior. This ambiguity highlights the need for field studies and ground truthing that can validate the radar observations. As previously mentioned, by increasing the temporal resolution of the dataset (i.e., aggregating the bat activity by nightly or weekly aggregations as opposed to the entire 2019 season) may provide valuable insights into the nuances of bat foraging over the course of time, especially when paired with the occurrences and abundances of various pests throughout the season. Ultimately, addressing these challenges will not only advance our understanding of bat ecology but also inform the quantification and management of ecosystem serviced provided by this important species.

## 5 Conclusion

Quantifying ecosystem service provision necessitates understanding where service providers are active. Here we illustrate how a novel approach to quantify bat activity may help to overcome this challenge and provide insights into where ecosystem services may be realized on real landscapes. We identify elevated bat activity over rice agriculture, as well as where aerial pest densities are predicted to be high in California's Northern Central Valley, suggesting a possible role for bats in agricultural pest control. Extending this approach to estimate the effect of bat activity more rigorously on pest or mosquito abundances, or pesticide use, in combination with field‐based validation, are important future directions.

## Author Contributions

**Brian Lee:** conceptualization (equal), data curation (supporting), formal analysis (equal), methodology (equal), project administration (equal), resources (equal), software (equal), writing – original draft (equal), writing – review and editing (equal). **Samantha Sambado:** conceptualization (equal), formal analysis (equal), investigation (equal), methodology (equal), project administration (lead), writing – original draft (equal), writing – review and editing (equal). **D. Nākoa Farrant:** conceptualization (equal), data curation (equal), formal analysis (equal), methodology (equal), project administration (equal), resources (equal), software (equal), writing – original draft (equal), writing – review and editing (equal). **Anna Boser:** conceptualization (equal), data curation (equal), formal analysis (equal), methodology (equal), resources (equal), software (equal), visualization (equal), writing – original draft (equal), writing – review and editing (equal). **Kacie Ring:** conceptualization (equal), formal analysis (equal), visualization (equal), writing – original draft (equal), writing – review and editing (equal). **David Hyon:** formal analysis (equal), methodology (equal), software (equal), writing – original draft (equal), writing – review and editing (equal). **Ashley E. Larsen:** conceptualization (equal), funding acquisition (equal), investigation (equal), methodology (equal), project administration (equal), supervision (equal), writing – original draft (equal), writing – review and editing (equal). **Andrew J. MacDonald:** conceptualization (equal), formal analysis (equal), investigation (equal), project administration (equal), software (equal), supervision (equal), writing – original draft (equal), writing – review and editing (equal).

## Conflicts of Interest

The authors declare no conflicts of interest.

## Supporting information


Data S1.

## Data Availability

Data sources and URLS used for the analysis are as follows: CA PUR, 2020 (https://apps.cdpr.ca.gov/docs/label/labelque.cfm); CDFA, 2020 (https://www.cdfa.ca.gov/Statistics/#:%E2%88%BC:text=California%20agricultural%20exports%20totaled%20%2421.7,%2C%20Davis%2C%20Agricultural%20Issues%20Cent); CALFIRE‐FRAP, 2015 (https://map.dfg.ca.gov/metadata/ds1327.htm l); Land IQ, 2019 (https://www.landiq.com/land‐use‐mapping); VectorSurv, 2023 (https://gateway.vectorsurv.org/), UCANR Pest Management (https://ipm.ucanr.edu/legacy_assets/pdf/pmg/pmgrice.pdf & https://ipm.ucanr.edu/agriculture/rice/armyworms/), UC Davis Army Worm Monitoring (https://agronomy‐rice.ucdavis.edu/armyworm‐monitoring). Additional details regarding data used for this study can be found in Appendix A SI.4. The R code used for land cover and mosquito analysis is available on GitHub at https://github.com/anna‐boser/larsen‐lab‐bats. The underlying bat activity data will be available on request.
