# Supplementary material for: Novel Bat‐Monitoring Dataset Reveals Targeted Foraging With Agricultural and Pest Control Implications
Source: Ecol Evol. 2025 Jan 15;15(1):e70819. doi: 10.1002/ece3.70819 (PMC11733310; doi:10.1002/ece3.70819)
Supplement: Supplementary file 1 — Data S1. [file ECE3-15-e70819-s001.docx]

**APPENDIX A: SUPPLEMENTAL INFORMATION (SI)**

**Title:** Novel bat-monitoring dataset reveals targeted foraging with agricultural and pest control implications

**Authors:** Brian Lee^1^*, Samantha Sambado^2^*, D. Nākoa Farrant^1^, Anna Boser^1^, Kacie Ring^2^, David Hyon^1^, Ashley E. Larsen^1^, and Andrew J. MacDonald^1^

1. Bren School of Environmental Science & Management, University of California Santa Barbara, CA, USA
2. Department of Ecology, Evolution, and Marine Biology, University of California, Santa Barbara, CA, USA

*Co-first authors

Corresponding author: [bhyleee@gmail.com](mailto:bhyleee@gmail.com); Bren Hall, 2400 University of California, Santa Barbara, CA 93117.

Supplemental Material

This file includes:

**SI.1:** Bat presence methods and figures

- Figure S1

**SI.2:** Landcover methods and figures

- Table S1
- Figure S2
- Figure S3
- Figure S4

**SI.3:** Mosquito abundance figures

- Figure S5
- Figure S6
- Figure S7

**S1.4:** Data descriptors and locators

- Table S2

**SI.1:** BAT PRESENCE ANALYSIS

Methods

*Post-processing of the data*

Additional post processing of the data includes identifying false positives and removing these pixels from the dataset. Due to the low-flying nature of foraging bats and our goal of identifying which crop types and land cover receive the most bat activity, we processed and classified radar data from the lowest elevation setting. Unfortunately, this results in many other objects being present in the radar data, which introduces the opportunity for false positive classification. These include different types of objects that the model falsely identifies as bats, including trains, large vehicles and traffic on raised highways, large buildings, wind turbines, and radar-facing hills and slopes. To remove these noisy features, we classified data from daytime scenes when bats are not active, created an aggregated mask layer, and removed false positive pixels from our analysis. Further algorithm details can be found in Lee et al. (2024).

*Spatial correction of the data*

This adjustment aimed to reduce the positive bias around roosts, thereby refining our estimates of bat activity based on foraging activity alone. We calibrated exponential decay functions for each of the eight roosts within our study area, applying these pixels within a 5 km radius of the roost. By summing the outcomes of these decay functions across the study area and adjusting for negative values resulting from overly rapid decay at the edges, we created a baseline expectation of bat presence based solely on roost location and size (**Fig. S1a**). However, we noted an overestimation of bat presence within 111 m of roosts by the exponential model. To correct from this, we excluded data from within this proximity. Our final step involved subtracting the expected bat presence derived from our model from the observed data, thereby obtaining a corrected distribution map. Negative values are associated with ‘No Data’ values and masked out, ensuring that our final map (**Fig. S1b**) accurately represents the adjusted foraging activity of bats across the landscape, which we will refer to as bat activity.

Figure


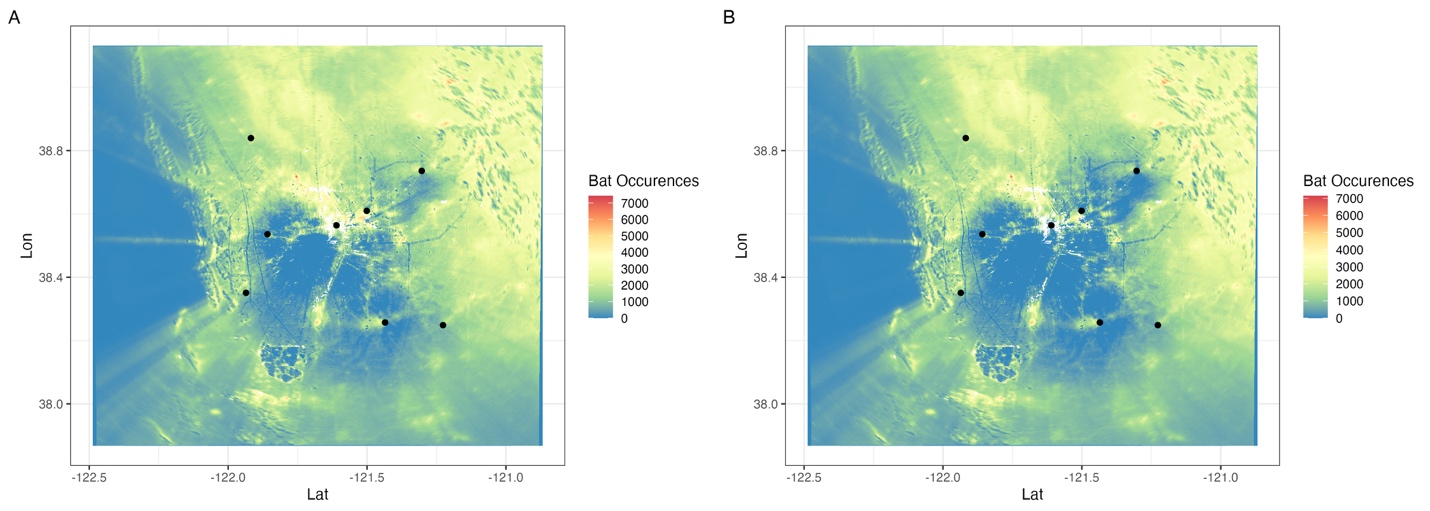


**Figure S1**. Spatial correction of bat activity over study area. (**a**)The original bat occurrence raster as processed using the BATS framework. (**b**) The exponential correction based on roost presence.

**SI.2:** LAND COVER ANALYSIS

Methods

*Combining two land cover datasets to produce comprehensive map layer*

After clipping both statewide datasets, FVEG and Land IQ, to the study region, the FVEG raster was resampled from 30 m to the resolution of the bat data layer (70 m). The resampled raster was vectorized based on the WHR10NAME category (**Table S1**). Land IQ croplands were erased from the vectorized FVEG layer. The result of the erasure was unioned with the Land IQ layer to create the combined land cover layer that prioritized the more recent and accurate Land IQ crop information where it was available, and FVEG data were used to label land cover at any locations not labelled as agriculture by Land IQ. The Land IQ layer specifies the outlines of fields, so there are residual areas identified as ‘Agriculture’ in 2015 but were not identified as fields in the 2019 Land IQ cropland dataset. These areas represent either fallow areas, roads between fields, or other areas zoned for agriculture that are not used for cultivation and were labeled as ‘Miscellaneous’ for this study. While it is possible that these areas stopped being used for agriculture since the 2015 FVEG layer was created, it is unlikely that areas labeled as ‘Miscellaneous’ have been heavily urbanized and likely represent semi-natural areas, agricultural facilities, or bare land covers. The land cover class names used in this study and their original names in their respective data sources are described in **Table S1**.

*Overlapping bat activity on new land cover layer*

Bat occurrences were extracted within each parcel of the final agricultural land vector layer. The area of each parcel was calculated, and bat presence per hectare was calculated within each parcel. Parcels were then grouped by each final land cover class (**Table S1**), and the area-weighted average value of bat occurrences per hectare associated with each land cover class was calculated. Area-weighted averages were used to adjust for substantial heterogeneity in parcel area size across many classes (**Fig. S2**). Any parcels that had no observed bat activity were assigned a value of zero bat occurrences per hectare and were included in the area-weighted average calculation.

The area-weighted average bat occurrences per hectare across the entire study area was separately calculated without grouping by land cover class.

Table and Figures

**Table S1**. Crosswalk between the original class names in the FVEG and Land IQ data sets and the final land cover class groupings used to assess bat presence over different land cover types. For the FVEG data set original classes were derived from the WHR10NAME attribute. For the Land IQ data, we used the CROPTYP2 attribute which corresponds to a crop type on single cropped fields.

| **Original Class Name** | **Column from Data Source** | **Data Source** | **Final Land Cover Class** |
| --- | --- | --- | --- |
| Agriculture | WHR10NAME | FVEG | Miscellaneous |
| Barren/Other | WHR10NAME | FVEG | Barren_Other |
| Conifer | WHR10NAME | FVEG | Conifer |
| Desert | WHR10NAME | FVEG | Desert |
| Hardwood | WHR10NAME | FVEG | Hardwood |
| Herbaceous | WHR10NAME | FVEG | Herbaceous |
| Shrub | WHR10NAME | FVEG | Shrub |
| Urban | WHR10NAME | FVEG | Urban |
| Water | WHR10NAME | FVEG | Water_Wetland |
| Wetland | WHR10NAME | FVEG | Water_Wetland |
| Citrus | CROPTYP2 | Land IQ | Fruits_Nuts_Vineyards |
| Dates | CROPTYP2 | Land IQ | Fruits_Nuts_Vineyards |
| Olives | CROPTYP2 | Land IQ | Fruits_Nuts_Vineyards |
| Misc. subtropical fruit | CROPTYP2 | Land IQ | Fruits_Nuts_Vineyards |
| Kiwi | CROPTYP2 | Land IQ | Fruits_Nuts_Vineyards |
| Apples | CROPTYP2 | Land IQ | Fruits_Nuts_Vineyards |
| Cherries | CROPTYP2 | Land IQ | Fruits_Nuts_Vineyards |
| Peaches and nectarines | CROPTYP2 | Land IQ | Fruits_Nuts_Vineyards |
| Pears | CROPTYP2 | Land IQ | Fruits_Nuts_Vineyards |
| Misc. deciduous | CROPTYP2 | Land IQ | Fruits_Nuts_Vineyards |
| Mixed deciduous | CROPTYP2 | Land IQ | Fruits_Nuts_Vineyards |
| Almonds | CROPTYP2 | Land IQ | Fruits_Nuts_Vineyards |
| Walnuts | CROPTYP2 | Land IQ | Fruits_Nuts_Vineyards |
| Pistachios | CROPTYP2 | Land IQ | Fruits_Nuts_Vineyards |
| Pomegranates | CROPTYP2 | Land IQ | Fruits_Nuts_Vineyards |
| Plums | CROPTYP2 | Land IQ | Fruits_Nuts_Vineyards |
| Cotton | CROPTYP2 | Land IQ | Row_Field |
| Safflower | CROPTYP2 | Land IQ | Row_Field |
| Beans | CROPTYP2 | Land IQ | Row_Field |
| Misc. field | CROPTYP2 | Land IQ | Row_Field |
| Sunflowers | CROPTYP2 | Land IQ | Row_Field |
| Corn | CROPTYP2 | Land IQ | Row_Field |
| Grain | CROPTYP2 | Land IQ | Row_Field |
| Wheat | CROPTYP2 | Land IQ | Row_Field |
| Misc. grain | CROPTYP2 | Land IQ | Row_Field |
| New crop land | CROPTYP2 | Land IQ | Row_Field |
| Alfalfa | CROPTYP2 | Land IQ | Grassland |
| Mixed pasture | CROPTYP2 | Land IQ | Grassland |
| Misc. grasses | CROPTYP2 | Land IQ | Grassland |
| Rice | CROPTYP2 | Land IQ | Rice |
| Wild Rice | CROPTYP2 | Land IQ | Rice |
| Truck and berries | CROPTYP2 | Land IQ | Row_Field |
| Onions and garlic | CROPTYP2 | Land IQ | Row_Field |
| Tomatoes | CROPTYP2 | Land IQ | Row_Field |
| Flowers and nursery | CROPTYP2 | Land IQ | Row_Field |
| Misc. truck | CROPTYP2 | Land IQ | Row_Field |
| Bush berries | CROPTYP2 | Land IQ | Row_Field |
| Strawberries | CROPTYP2 | Land IQ | Row_Field |
| Peppers | CROPTYP2 | Land IQ | Row_Field |
| Greenhouse | CROPTYP2 | Land IQ | Barren_Other |
| Leafy greens | CROPTYP2 | Land IQ | Row_Field |
| Potato and sweet potato | CROPTYP2 | Land IQ | Row_Field |
| Cole crops | CROPTYP2 | Land IQ | Row_Field |
| Carrots | CROPTYP2 | Land IQ | Row_Field |
| Melons and squash | CROPTYP2 | Land IQ | Row_Field |
| Urban | CROPTYP2 | Land IQ | Urban |
| Vineyards | CROPTYP2 | Land IQ | Fruits_Nuts_Vineyards |
| Not cropped | CROPTYP2 | Land IQ | Barren_Other |
| No subclass | CROPTYP2 | Land IQ | Barren_Other |

**
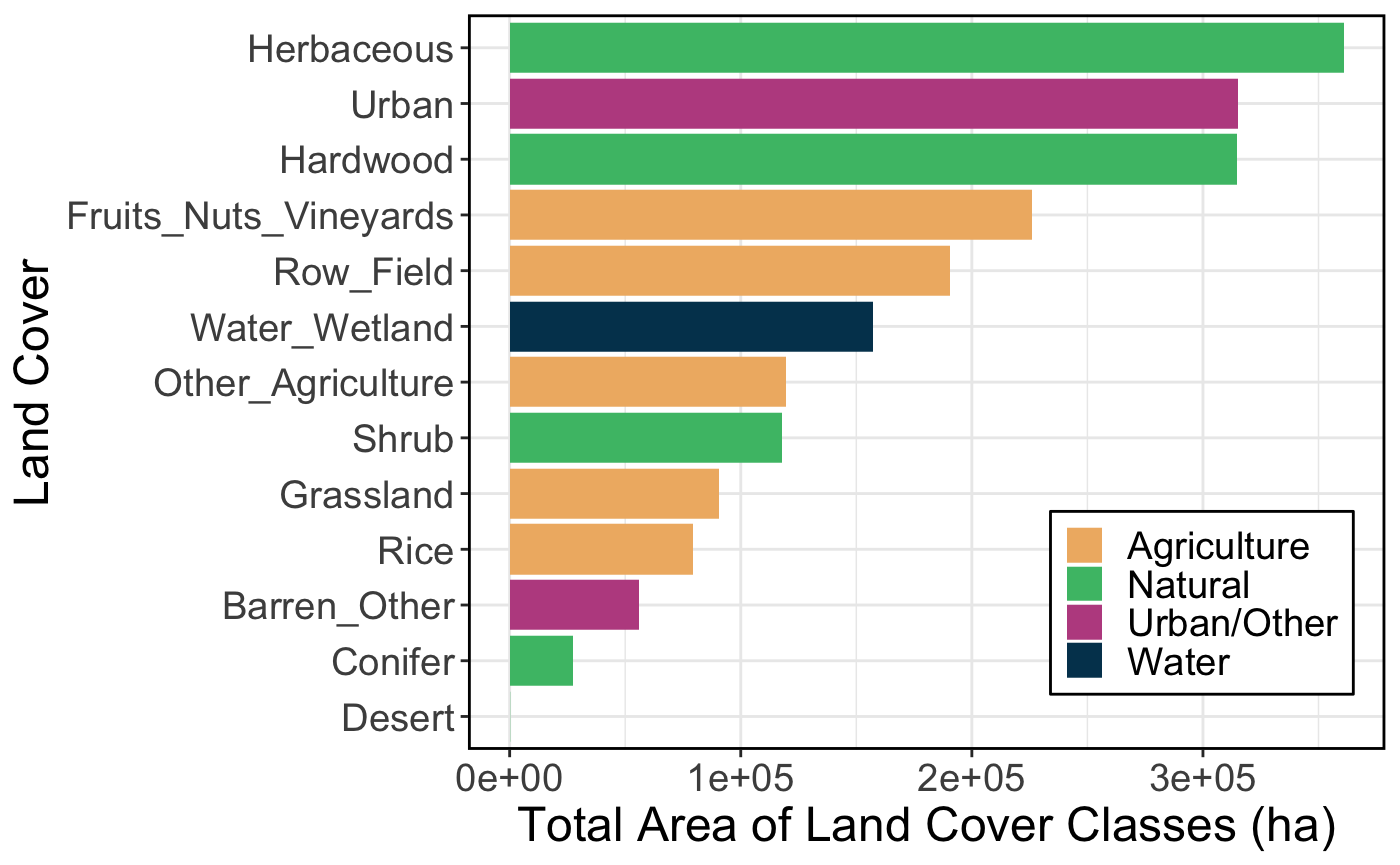
**

**Figure S2**. Area of land cover categories in the study region based on a combination of FVEG and LandIQ data (see **Table S1**). Other agriculture refers to miscellaneous land cover type is primarily areas that ‘FVEG’ identified as agriculture in 2015 but were not labeled as cropland in the more accurate cropland data from 2019. The miscellaneous land cover includes field margins as well as areas that were potentially fallowed or used for other agricultural purposes.

**
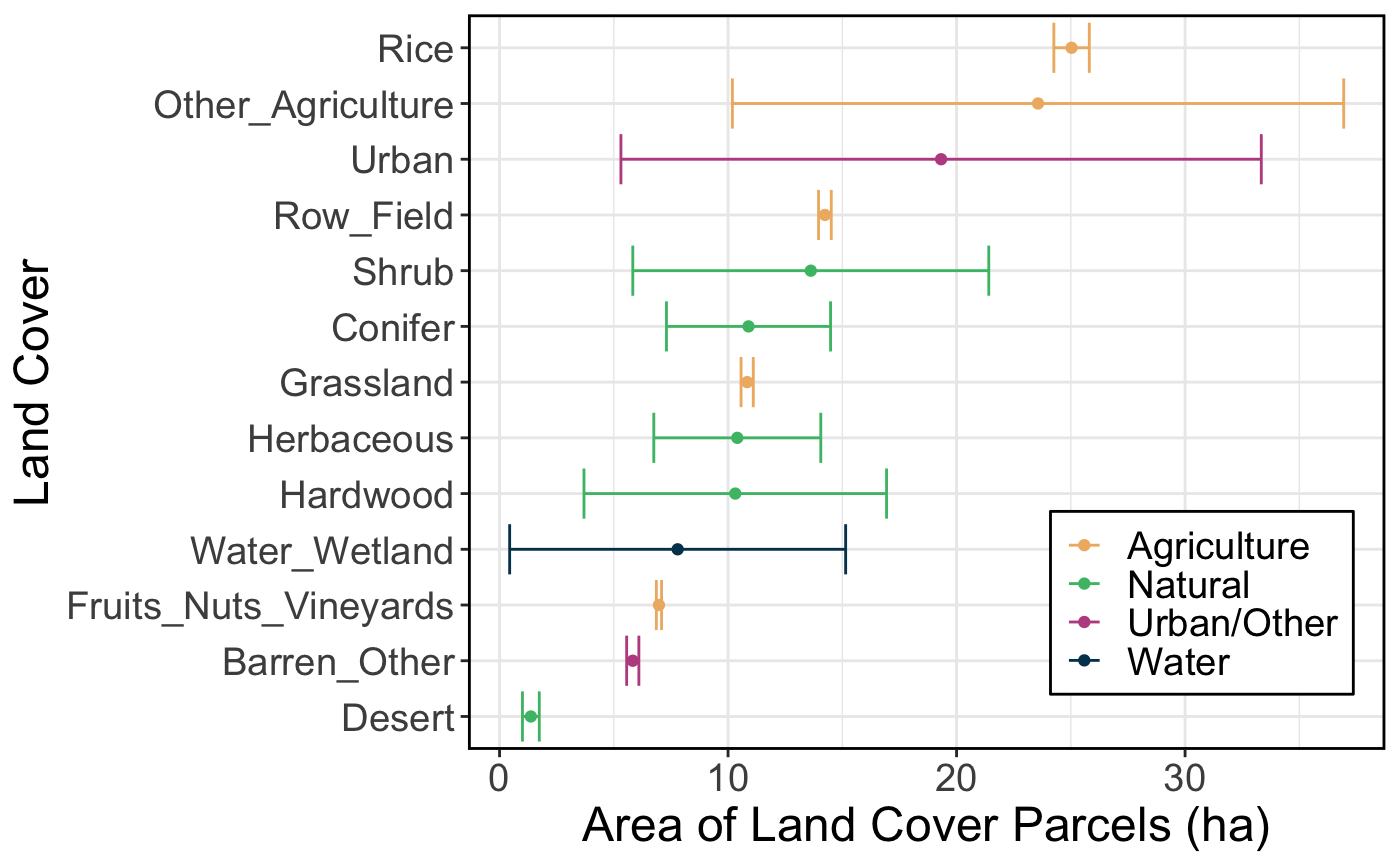
**

**Figure S3**. Average area of parcels in each land cover type in the study region. Rice has the highest average parcel area. ‘Roads and Misc. Ag. Land’ includes areas labeled as agricultural land in FVEG but encompasses roads between fields identified in the more granular Land IQ dataset that identifies individual field boundaries. This land use category may also include lands that were fallowed since FVEG was created in 2015. Roads and miscellaneous agricultural land and urban land covers had the next highest average parcel sizes, though the parcel area for both land cover types varied considerably. Each point represents the average area of a parcel or fragment in each land cover category and error bars represent the 95% confidence interval.

**
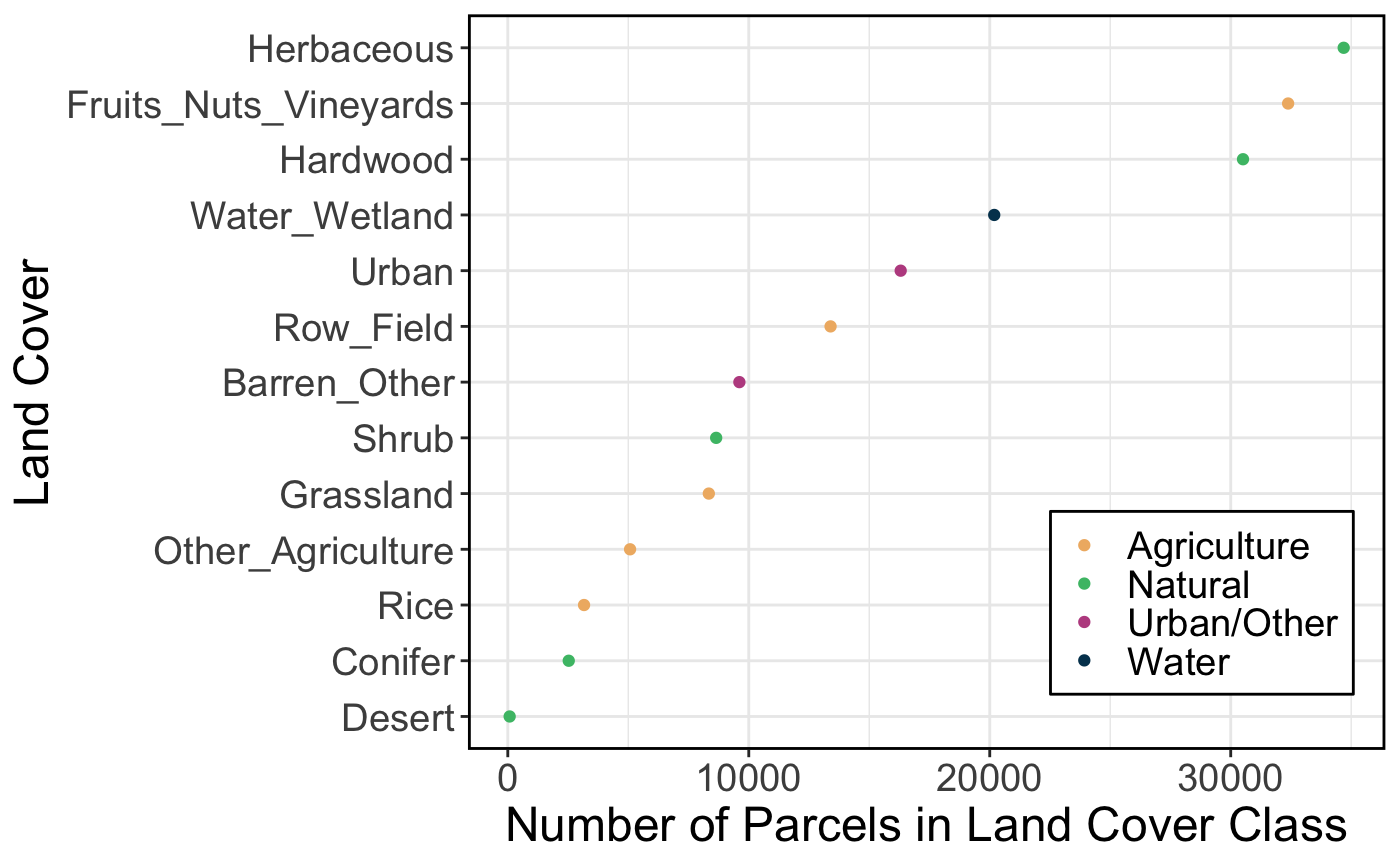
**

**Figure S4**. Number of parcels in each land cover type. Each point represents the total number of parcels in each land cover type in the study area. Other agriculture refers to miscellaneous land cover type is primarily areas that ‘FVEG’ identified as agriculture in 2015 but were not labeled as cropland in the more accurate cropland data from 2019. The miscellaneous land cover includes field margins as well as areas that were potentially fallowed or used for other agricultural purposes.

**SI.3:** MOSQUITO ANALYSIS

Figures

**
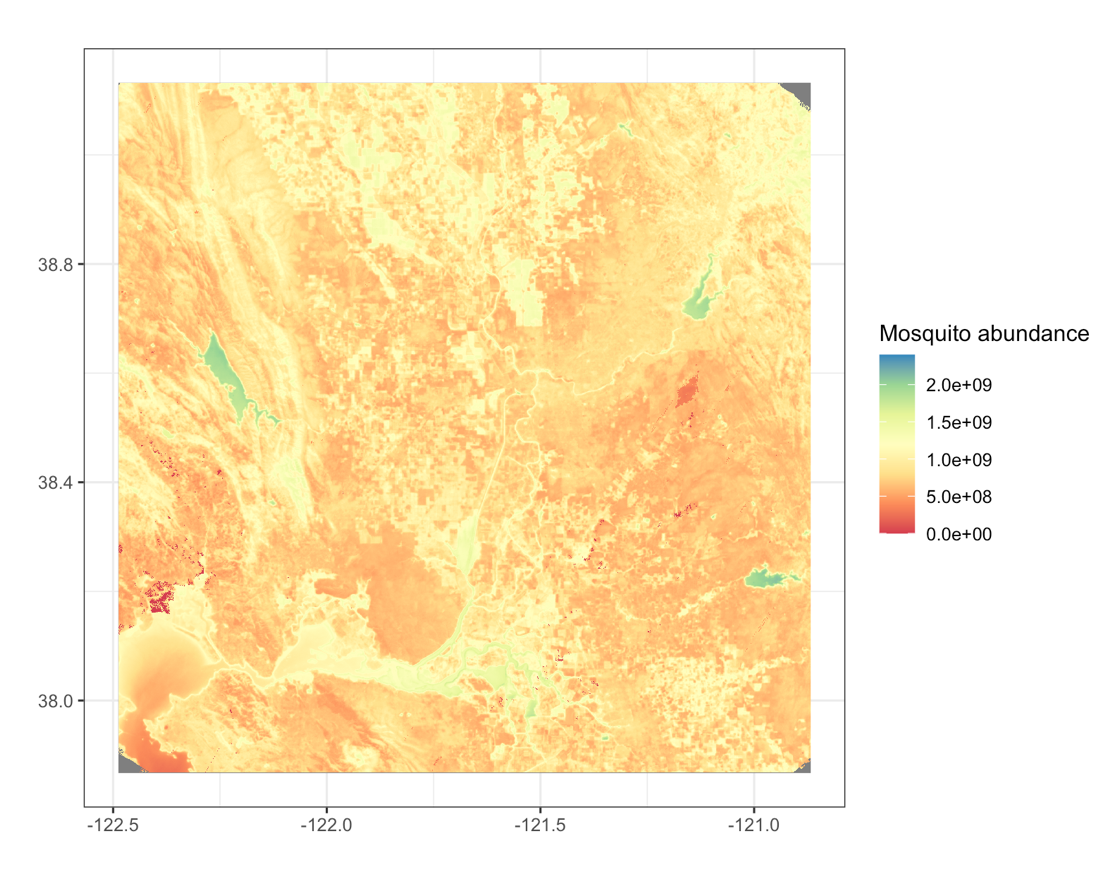
**

**Figure S5**. Modeled temperature-dependent adult female *Culex tarsalis* abundance across study area.

**
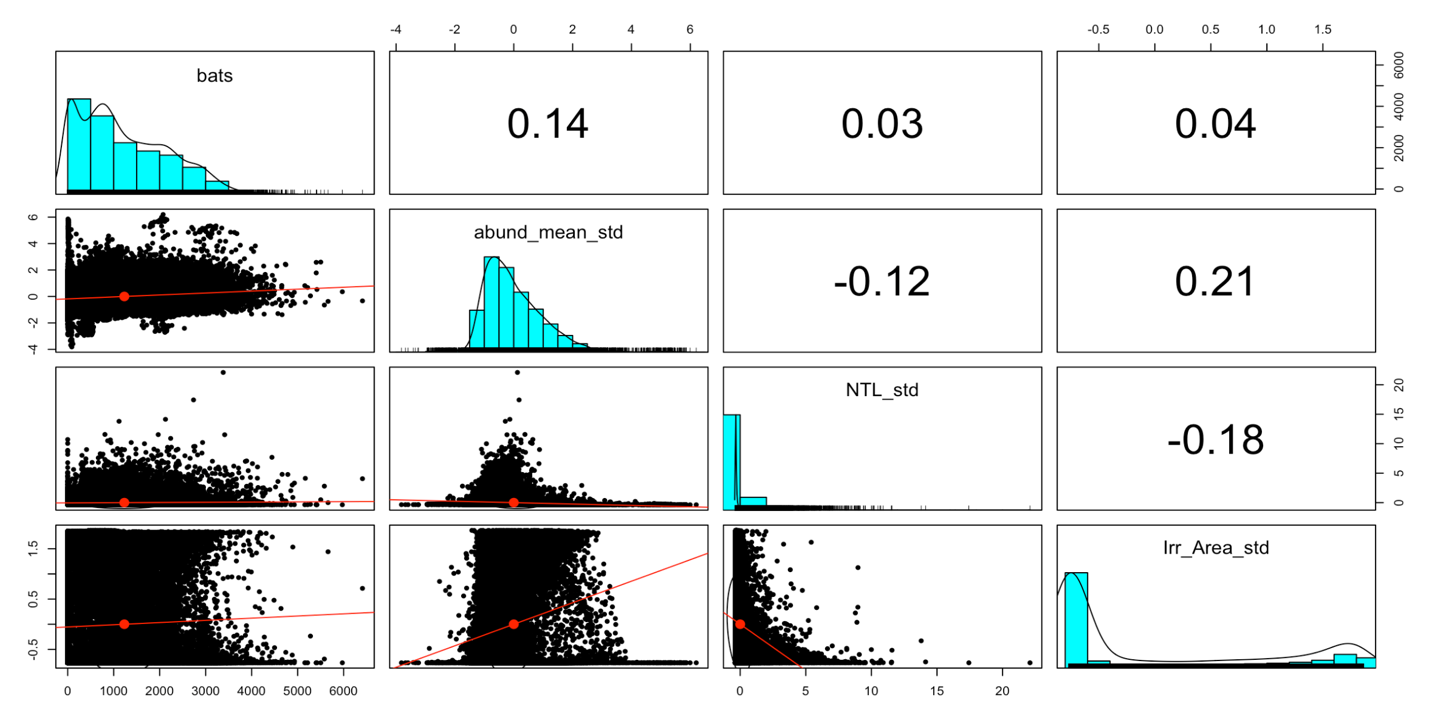
**

**Figure S6**. Correlation plot of generalized additive model (GAM) variables.

**
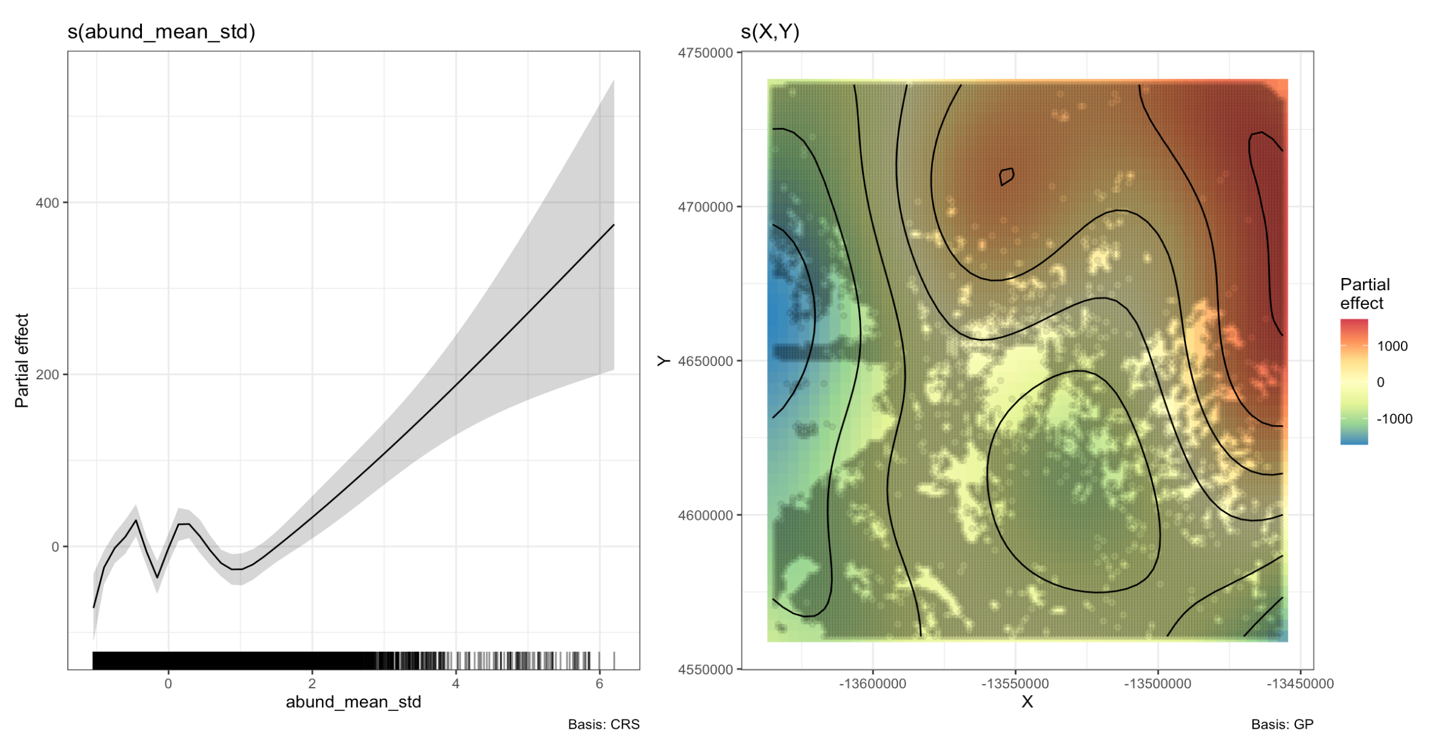
**

**Figure S7**. Estimated (**a**) smooth and (**b**) bivariate smooths from generalized additive model GAM.

**SI.4:** DATA DESCRIPTORS AND LOCATORS

**Table 2**. Summary of data sources for data type and method sections.

| **Method Section** | **Data Type** | **Source** | **Hyperlink** |
| --- | --- | --- | --- |
| **2.2 Bat Presence** | Bat activity | NOAA NEXRAD Big Data Project | <https://cloud.google.com/storage/docs/public-datasets/nexrad> |
| **2.3 Land Cover Analysis** | Natural Land cover | CALFIRE-FRAP | <https://map.dfg.ca.gov/metadata/ds1327.html> |
|  | Cropland | Land IQ | <https://www.landiq.com/land-use-mapping> |
| **2.4-5 West Nile Estimates** | Mosquito abundance | VectorSurv | <https://gateway.vectorsurv.org/> |
|  | WNV transmissibility | ECOSTRESS | <https://appeears.earthdatacloud.nasa.gov/> |
|  | Night light emissions | Google Earth Engine | <https://developers.google.com/earth-engine/datasets/catalog/NOAA_DMSP-OLS_NIGHTTIME_LIGHTS> |
|  | Area of irrigated water | Google Earth Engine | <https://developers.google.com/earth-engine/datasets/catalog/UMT_Climate_IrrMapper_RF_v1_1> |
